# Editorial: The Gating and Maintenance of Sleep and Wake: New Circuits and Insights

**DOI:** 10.3389/fnins.2020.00773

**Published:** 2020-08-11

**Authors:** Michael Lazarus, Yu Hayashi, Sakiko Honjoh, Kaspar E. Vogt, Ada Eban-Rothschild, Qinghua Liu, Takeshi Sakurai

**Affiliations:** ^1^International Institute for Integrative Sleep Medicine (WPI-IIIS), University of Tsukuba, Tsukuba, Japan; ^2^Graduate School of Medicine, Kyoto University, Kyoto, Japan; ^3^Department of Psychology, University of Michigan, Ann Arbor, MI, United States; ^4^National Institute of Biological Sciences, Beijing, China; ^5^Tsinghua Institute of Multidisciplinary Biomedical Research, Tsinghua University, Beijing, China

**Keywords:** sleep, sleep need, arousal, sleep-wake cycle, wakefulness

Sleep is highly conserved among all organisms with a nervous system, from worms to humans, and is vital for survival (Siegel, 2008; Trojanowski and Raizen, 2016). Humans spend one-third of their lives asleep, but due to work schedules and expectations, life-style choices, or medical conditions, many people experience a wide range of sleep disturbances (Kryger et al., 2017). Sleep disturbances may take a serious social and economic toll due to an increased prevalence of psychiatric illnesses, especially anxiety and mood disorders, decreased economic productivity, and a strong link to traffic and work-related accidents. Insufficient sleep is also an established risk factor for obesity, diabetes, heart disease, and other lifestyle diseases, as well as infectious diseases and cancers.

The governing principles of sleep remain one of the biggest black boxes of neuroscience. Therefore, there is increasing urgency to gain knowledge of the neural mechanisms and molecular substrates that regulate sleep and to reveal the causal links between sleep and health. New opto-/chemo-genetic-based tools and tracing methods for neural circuits have enabled unprecedented investigation of discrete circuit elements (e.g., transmitters, pathways, cell populations) and identification of genes and signaling pathways that regulate sleep/wake behavior (Saper et al., 2010; Saper and Fuller, 2017). As a result, new cellular and molecular targets for treating sleep disorders have been identified (Saper and Scammell, 2013). In this Research Topic, we present up-to-date original articles and reviews that demonstrate the versatility and power of state-of-the-art tools in advancing our knowledge of the regulation and function of sleep.

The neurotransmitter serotonin is believed to play an important role in sleep/wake regulation, but the underlying molecular mechanisms and brain circuits remain unclear. Iwasaki et al. used diptheria toxin-mediated ablation of central serotonergic neurons to examine the role of serotonergic neurons in the sleep/wake behavior of adult mice. Their findings suggest that serotonergic neurons support wakefulness and regulate REM sleep through a biased transition from non-rapid eye movement (non-REM, NREM) sleep to REM sleep. In addition, Saito et al. examined the physiologic relevance of serotonergic regulation of orexin neurons in mice lacking inhibitory serotonin 1A receptors, specifically in orexin neurons. They found that serotonin regulates the orexinergic tone that is important for maintaining the sleep/wake architecture. Although GABAergic neurons in the parafacial zone are implicated in NREM sleep (also known as slow-wave sleep), the function of glutamatergic neurons in this brain area is unknown. Therefore, Erickson et al. utilized a chemogenetic approach to investigate the role of glutamatergic neurons in the parafacial zone in regulating NREM sleep in mice.

One advantage of using transgenic animals is to simplify the identification of neuronal populations and their projections, which can be laborious using traditional techniques. Yuan et al. employed rabies virus-based retrograde tracing to map and quantitatively analyze the whole-brain monosynaptic inputs to cholecystokinin-expressing neurons in the suprachiasmatic nucleus. This research is complemented by the article by Chen et al., as they characterized the monosynaptic inputs and axonal projections of GABAergic neurons in the lateral pontine tegmentum, which play key roles in regulating sleep and locomotion, using virally mediated, cell-type-specific, retrograde and anterograde tracing systems.

*In-vivo* imaging used in combination with pharmacologic or genetically driven perturbation of defined sets of neurons or glial cells is useful for identifying the brain circuits underlying sleep regulation. For example, Dong et al. employed a genetically encoded dopamine indicator to track striatal dopamine levels across the sleep-wake cycle and in response to external stimuli. In addition, Patel et al. describe simultaneous electrophysiologic recording and cell-type-specific imaging for interrogating state-dependent neural circuit dynamics *in vivo*. To bridge the gap in our knowledge about the dynamic changes in neurotransmitter systems during the transition from wake to sleep in humans,  Lehmann et al. utilized functional magnetic resonance spectroscopy in combination with polysomnography to detect naturally occurring thalamic metabolite concentrations, a methodology that may hold potential for investigating the molecular mechanisms underlying the transition between sleep and wake states and the maintenance of these states in humans. Moreover, human sleep studies may also benefit from the automated classification of sleep stages based on machine-learning algorithms, as, for example, described in the research article by Malafeev et al..

All living organisms respond to stress, i.e., perceived or actual threats, with a predictable biologic pattern in an attempt to restore body homeostasis. Stress is a risk factor for mental disorders and often leads to sleep alterations. Fujii et al. established a mouse model of acute social defeat stress based on a resident-intruder paradigm and examined its effects on the sleep/wake behavior of the mice. The authors demonstrated that NREM sleep strongly increases in response to social defeat stress. Although some of the described sleep changes can be attributed to non-specific effects of the social defeat procedure, most of the NREM sleep increase is likely a specific stress response. By contrast, Yasugaki et al. investigated how sleep changes during a long period of chronic stress in a mouse model of depression induced by water immersion and restraint stress. The authors report that chronic stress produced in their mouse model differentially affects sleep in early and subsequent periods. Likewise, Lou et al. studied changes in the sleep-wake architecture of mice exposed to a single episode of prolonged stress. The authors report a causal link between persistent activation of the mouse medial prefrontal cortex and specific short/long-term electroencephalogram alterations induced by a single episode of prolonged stress.

Insomnia is a subjective complaint of inadequate sleep and the most frequently prescribed hypnotics to treat insomnia are benzodiazepines, which are unsafe when excessively dosed or used for long-term treatment due to severe side effects such as addiction and interference with memory processes (Brett and Murnion, 2015). Benzodiazepines also increase the risk of falling in the elderly (Díaz-Gutiérrez et al., 2017). Several papers in this Research Topic evaluated alternative pharmacologic strategies for the treatment of insomnia. For example, Feng et al. reported that oral delivery of the highly selective α2-adrenergic agonist dexmedetomidine can induce sedative and hypnotic effects. In addition, Yoon et al. report that dieckol, a polyphenol from the brown alga species *Ecklonia cava* found off the coasts of Japan and Korea, promotes sleep via the benzodiazepine binding site of the GABA_A_ receptor in mice. These articles are complemented by the study from Cherasse et al. examining the sleep-inducing effect of prostaglandin D_2_ in mice.

Additional original papers examined different aspects of electrocortical activity during sleep and wakefulness. Specifically, Tavakoli et al. present evidence for the occurrence of P3a, a component of event-related potentials elicited by auditory stimuli during sleep in humans, whereas evidence provided by Carrera-Cañas et al. may indicate the existence of a transition state between NREM and REM sleep in rats. The Research Topic is further enriched by a study from Nam et al. on transcriptome analysis of the pineal gland in a mouse model of Alzheimer disease. As the hormone melatonin, which is responsible for regulating the sleep/wake cycle, is produced in the pineal gland of the brain, transcriptomic changes in the pineal glands of patients with Alzheimer disease may contribute to the sleep alterations that often accompany this neurodegenerative disease.

This Research Topic also contains a series of excellent authoritative reviews on various aspects of sleep/wake regulation and related topics. For example, a unified model accounting for the myriad of behavioral state-dependent changes in physiology, including the regulated reduction in body temperature that occurs with sleep, remains lacking. In this context, we highly recommend reading the thoughtful review by Harding et al. on the link between sleep and body temperature. Moreover, the sleep field has recently witnessed an exponential increase in the understanding of brain circuitries regulating sleep/wake behavior. On the other hand, it remains puzzling that brain circuits' switching occurs within seconds, while sleep regulation, i.e., the process of accumulation/dissipation of sleep need, takes hours. Obviously, there is a lack of sufficient experimental data for the integration of glia-neuron interactions, brain circuit activity, and intracellular signal transduction. In this context, Kaur and Saper provide an overview on the circuitries that regulate waking-up from sleep in response to hypercapnia, and Mieda discusses the different roles of multiple neuropeptides and neuropeptide-expressing neurons in the suprachiasmatic nucleus, the central circadian pacemaker in mammals. These “circuit/network” reviews are complemented by two perspective reviews on the integration of neuronal and molecular mechanisms in the regulation of sleep. Yamada and Ueda provide a fresh look at REM sleep regulation from a molecular point of view, whereas Lazarus et al. review the various roles of the classic somnogen adenosine in gating sleep and regulating sleep need.

In summary, the complexity of sleep control by the nervous system and the mystery surrounding the functions of sleep remain a subject of great fascination among scientists. In an effort to tackle these questions, sleep studies are taking advantage of transgenic technologies, *in-vivo* recording/imaging of neural activity, “omics” approaches, and human functional magnetic resonance imaging. Emerging technologies, such as single-cell and spatial transcriptomics (Hammond et al., 2019; Maniatis et al., 2019) or *in-vivo* quantification of dynamic changes in neurotransmitters and neuromodulators (Sun et al., 2018; Feng et al., 2019) will further expand the sleep scientists' toolbox. We hope the articles included in this Research Topic will spark new ideas in laboratories that are interested in the sleeping, waking, and dreaming brain.

## Author Contributions

All authors listed have made a substantial, direct and intellectual contribution to the work, and approved it for publication.

## Conflict of Interest

The authors declare that the research was conducted in the absence of any commercial or financial relationships that could be construed as a potential conflict of interest.

## References

[B1] Brett J. Murnion B. (2015). Management of benzodiazepine misuse and dependence. Aust. Prescrib. 38, 152–155. 10.18773/austprescr.2015.055 26648651 PMC4657308

[B2] Díaz-Gutiérrez M. J. Martínez-Cengotitabengoa M. Sáez De Adana E. Cano A. I. Martínez-Cengotitabengoa M. T. Besga A.. (2017). Relationship between the use of benzodiazepines and falls in older adults: a systematic review. Maturitas 101, 17–22. 10.1016/j.maturitas.2017.04.002 28539164

[B3] Feng J. Zhang C. Lischinsky J. E. Jing M. Zhou J. Wang H.. (2019). A genetically encoded fluorescent sensor for rapid and specific *in vivo* detection of norepinephrine. Neuron 102, 745–761.e748. 10.1016/j.neuron.2019.02.037 30922875 PMC6533151

[B4] Hammond T. R. Dufort C. Dissing-Olesen L. Giera S. Young A. Wysoker A.. (2019). Single-cell RNA sequencing of microglia throughout the mouse lifespan and in the injured brain reveals complex cell-state changes. Immunity 50, 253–271.e256. 10.1016/j.immuni.2018.11.004 30471926 PMC6655561

[B5] Kryger M. H. Roth T. Dement W. C. (2017). Principles and Practice of Sleep Medicine, 6th Edn. Philadelphia, PA: Elsevier Health Sciences.

[B6] Maniatis S. Äijö T. Vickovic S. Braine C. Kang K. Mollbrink A.. (2019). Spatiotemporal dynamics of molecular pathology in amyotrophic lateral sclerosis. Science 364, 89–93. 10.1126/science.aav9776 30948552

[B7] Saper C. B. Fuller P. M. (2017). Wake-sleep circuitry: an overview. Curr. Opin. Neurobiol. 44, 186–192. 10.1016/j.conb.2017.03.021 28577468 PMC5531075

[B8] Saper C. B. Fuller P. M. Pedersen N. P. Lu J. Scammell T. E. (2010). Sleep state switching. Neuron 68, 1023–1042. 10.1016/j.neuron.2010.11.032 21172606 PMC3026325

[B9] Saper C. B. Scammell T. E. (2013). Emerging therapeutics in sleep. Ann. Neurol. 74, 435–440. 10.1002/ana.24000 24038193

[B10] Siegel J. M. (2008). Do all animals sleep? Trends Neurosci. 31, 208–213. 10.1016/j.tins.2008.02.001 18328577 PMC8765194

[B11] Sun F. Zeng J. Jing M. Zhou J. Feng J. Owen S. F.. (2018). A genetically encoded fluorescent sensor enables rapid and specific detection of dopamine in flies, fish, and mice. Cell 174, 481–496.e419. 10.1016/j.cell.2018.06.042 30007419 PMC6092020

[B12] Trojanowski N. F. Raizen D. M. (2016). Call it worm sleep. Trends Neurosci. 39, 54–62. 10.1016/j.tins.2015.12.005 26747654 PMC4738085

